# Investigation on Capacitance Collapse Induced by Secondary Capture of Acceptor Traps in AlGaN/GaN Lateral Schottky Barrier Diode

**DOI:** 10.3390/mi13050748

**Published:** 2022-05-09

**Authors:** Haitao Zhang, Xuanwu Kang, Yingkui Zheng, Ke Wei, Hao Wu, Xinyu Liu, Tianchun Ye, Zhi Jin

**Affiliations:** 1 High-Frequency High-Voltage Device and Integrated Circuits Center, Institute of Microelectronics of Chinese Academy of Sciences, Beijing 100029, China; zhanghaitao19@mails.ucas.edu.cn (H.Z.); zhengyingkui@ime.ac.cn (Y.Z.); weike@ime.ac.cn (K.W.); wuhao@ime.ac.cn (H.W.); xyliu@ime.ac.cn (X.L.); tcye@ime.ac.cn (T.Y.); 2 School of Electronic Electrical and Communication Engineering, University of Chinese Academy of Sciences, Beijing 100049, China; 3 The Institute of Future Lighting, Academy for Engineering and Technology, Fudan University (FAET), Shanghai 200433, China

**Keywords:** GaN, SBD, Schottky barrier diode, simulation, capacitance collapse, acceptor trap, potential, depletion, secondary capture

## Abstract

In this study, a dedicated dynamic measurement system was used to investigate the transient capacitance and recovery process of AlGaN/GaN lateral Schottky barrier diodes (SBDs). With the consideration of acceptor traps in the C-doped buffer, the C-V characteristics and transient capacitance were measured and analyzed, and the results were simulated and explained by Silvaco TCAD (technology computer aided design). The ionization of acceptor traps and the change of electric potential were monitored in transient simulation to investigate the origin of the capacitance collapse in the SBD. The results suggest the significant impact of traps in the GaN buffer layer on the capacitance collapse of the device, and the secondary capture effect on the variation of acceptor ionization. Based on the study of transient capacitance of SBD, this work could be extended to the Miller capacitance in high electron mobility transistor (HEMT) devices. Moreover, the report on the stability of capacitance is essential for GaN devices, and could be further extended to other aspects of device research.

## 1. Introduction

Gallium nitride devices are widely used in radio frequency (RF) and power switching circuits. However, parasitic capacitance in gallium nitride devices is problematic in its further improvements [1,2]. Here, we focus on the capacitance of thin-barrier AlGaN/GaN heterojunction Schottky barrier diodes (SBDs). It can be equivalently viewed as a capacitance between gate and drain (C_gd_) in high electron mobility transistor (HEMT) devices if adding an extra ohmic cathode. C_gd_ refers to the Miller capacitance of HEMT devices [3]. For power devices and RF devices, C_gd_ is the most important parasitic capacitance parameter [4]. The instability of C_gd_ will significantly affect the performance and stability of the equipment, and the change of capacitance of RF devices is directly related to the impedance matching of the circuit. If the capacitance value considerably fluctuates with RF electrical stress, it will result in change in RF circuit bandwidth, insertion loss and other problems [5,6,7]. The stability of capacitance is very important for gallium nitride devices. The existing body of research has recognized the degradation phenomenon after stressing during the RF operation, but no clear relationship has been established between device degradation, trapping effect and capacitance, and the degradation mechanism still remains problematic [8,9].

In this work, we mainly focus on the capacitance of thin-barrier AlGaN/GaN heterojunction Schottky barrier diodes. The ultra-thin barrier AlGaN and the ultra-thin SiN dielectric could substantially improve the capacitance caused by the field plate ([10], pp. 202–206). The change effect of capacitance is amplified by increasing the capacitance ratio brought by field plate, and the change of capacitance after stress could be well observed.

In our previous work [11,12,13], a model of an ultra-thin barrier gallium nitride SBD device with improved accuracy is carried out. In particular, the current collapse is studied in detail. In this paper, we firstly propose the concept of “capacitance collapse”. Different from the current collapse test, capacitance collapse monitors the capacitance value before and after the electrical stress instead of current monitoring. Therefore, capacitance collapse tests cannot be performed with conventional equipment. An AccoTEST STS8200 tester has been modified to quickly switch between capacitance monitoring and reverse high voltage stress.

The curve of capacitance collapse is well fitted with the correction from the simulation model. By studying the ionization of the acceptor traps and potential changes in the corrected model, the origin of capacitance collapse is discussed. The ionization of acceptor traps leads to the change of potential in GaN, leading to a changing depletion layer width in GaN and thus the change of capacitance. The secondary capture effect results in different levels of acceptor ionization. This study provides a new idea for the research of capacitance of GaN devices, and it can be further extended to other aspects of device research.

## 2. Device Characterization and Simulation

**Figure 1:**
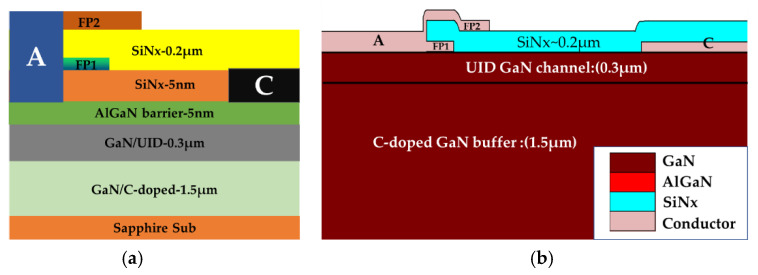
(**a**) Schematic of the Schottky barrier diode (SBD) for device under tests. (**b**) Schematic of the SBD from TCAD simulation.

The schematic of the SBD is shown in Figure 1. Metal organic chemical vapor deposition (MOCVD) was used to deposit the AlGaN/GaN heterostructure on 4-inch sapphire substrate. The smooth and crack-free epitaxial layer consists of a carbon-doped GaN buffer layer, a GaN channel, an AlN interface enhancement layer and a Al_0.25_Ga_0.75_N barrier with thicknesses of 1.5 μm/300 nm/1 nm and 5 nm. Two layers of field plates, namely FP1 and FP2, were progressively deposited with a 5 nm pre-grown LPCVD SiNx and a 200 nm interlayer SiNx.

**Figure 2:**
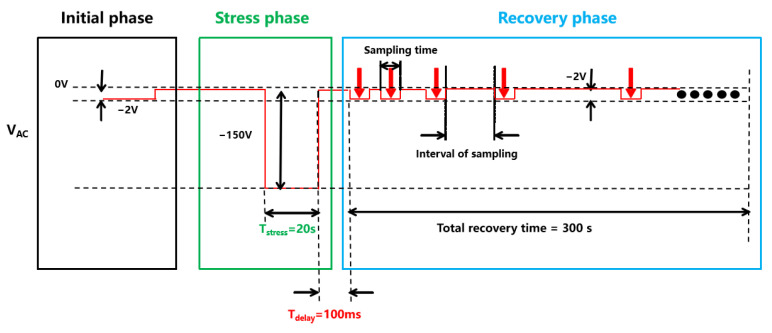
Dynamic measurement test sequence setting with STS8200.

The dynamic measurement sequence is summarized in Figure 2. At the initial phase, the initial capacitance (C_CA_) was measured as fabricated with an STS8200 at V_AC_ = −1.8 V and f = 1 MHz. During the second phase, the stress phase, a stress voltage (V_AC_) of −150 V was applied with a total stress time (t_stress_) of 20 s. After stressing, the recover capacitance C_CA_ (at V_AC_ = −1.8 V and f = 1 MHz) was measured immediately with a delay time (t_delay_) of 100 ms in the recovery phase. The interval between each sampling period was gradually increased with a total recovery time of 300 s. The dynamic characteristic simulation and the electrical measurement only differ in the constant V_AC_ of −1.8 V and frequency of 1 MHz in the recovery phase.

Previously, we have discussed the main simulation key parameters in detail [13]. In this study, we adjusted the thickness of SiN from 10 nm to 5 nm in order to obtain more observable capacitance changes. Compared with our previous work, we improved and optimized the simulation model to minimize its discrepancy with our device structure, especially the morphology of the field plate, in order to guarantee the accuracy of the capacitance simulation. Some simulation key parameters have been fine-tuned: the energy level of acceptor traps (E_TA_) in C-doped buffer is adjusted to E_V_ +0.95 eV and the donor trap energy level (E_TD_) is adjusted to E_C_ −1.05 eV. The energy level of GaN is measured to be E_C_ −1.10 eV in existing body of research [14], and the energy level of carbon doping of E_V_ +0.9 eV has been extensively confirmed in previous studies [15,16,17,18,19,20,21,22]. The electrical measurements fit the simulation results well. Some simulated key parameters are summarized in Table 1.

**Figure 3:**
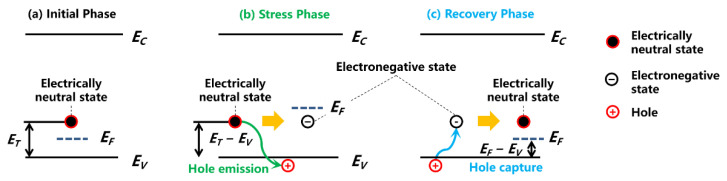
Schematic of acceptor-trap-induced capture and emission of free carriers.

Through the capture and emission of free carriers, the charge trapping effects can affect the electrical potential inside the device. For instance, as shown in Figure 3b, the deep-level acceptor traps in the GaN buffer layer (mainly in the C-doped region) would release holes under a high electric field, yielding negative space charges in the buffer layer [23,24]. These negative space charges would result in the reduction of the potential of C-doped GaN buffer layer. When entering the recovery phase, these negatively charged acceptor traps accumulating in GaN buffer start to capture holes from the valence band, recovering into an electrically neutral state, as shown in Figure 3c.

## 3. Results and Discussion

**Figure 4:**
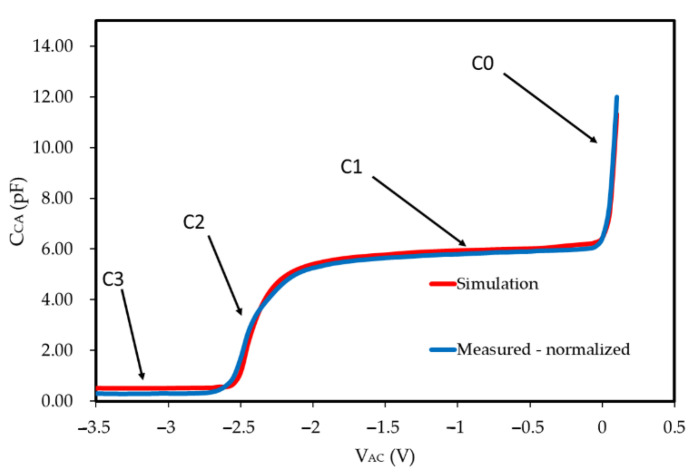
Capacitance-voltage characteristics of measurement and simulations.

The C-V curve measurement was carried out on GaN SBDs with L_AC_ = 6 μm. As could be observed from Figure 4, the measured and normalized C-V curve (in blue) relatively matches the simulated C-V curve (in red). The actual test capacitance is larger than the simulated capacitance because of the existence of auxiliary regions contributing to parasitic capacitance in the actual device structure. We normalized the actual capacitance according to the capacitance result at V_AC_ = 0 V and the capacitance of the subsequent dynamic test. C0 is the depletion capacitance of 2DEG, C1 is the capacitance of FP, C2 is the depletion process of the 2DEG under FP, C3 is the drift region capacitance. As V_AC_ becomes negative, the 2DEG below the anode is depleted. V_AC_ continued to become negative and the capacitance of FP was depleted. As shown in Figure 4, the capacitance changes of the simulation and actual test maintain the same trend. There is a certain deviation between the simulation and the actual test, which is mainly caused by the differences in the doping concentration, dielectric thickness and polarization coefficient between the actual and simulated devices.

**Figure 5:**
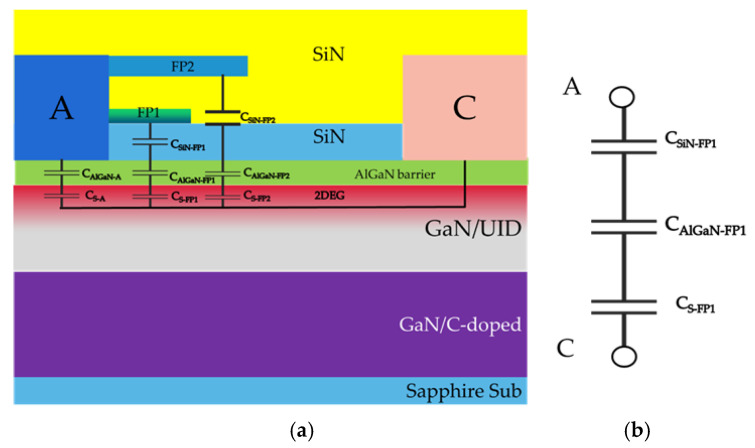
(**a**) Parasitic capacitance model of device. (**b**) Equivalent circuit model.

Figure 5 presents the parasitic capacitance model of the device and its equivalent circuit in reverse bias state (V_AC_ = −1.8 V). The capacitance related to SiN and AlGaN is considered as a dielectric capacitance and is related to the dielectric constant, area and thickness of the dielectric. As could be observed from Figure 4, the 2DEG under anode metal has been depleted in the state of reverse bias. Therefore, the capacitance associated with the anode region can be ignored. Since the medium under FP2 is thicker, the capacitance of the field plate can only be equivalent to that of FP1. The change of device capacitance in reverse bias state (V_AC_ = −1.8 V) is mainly influenced by the variation of the depletion layer in UID-GaN. C_CA_ decreases with the increase of V_CA_, mainly because the high reverse bias would extend the depletion region, leading to the change of capacitance.

The capacitance of semiconductor devices in the depleted state could be estimated with the following relationship ([10], pp. 197–240):

(1) $$C_{s}=\frac{\varepsilon_{s}\varepsilon_{0}}{\sqrt{2}L_{D}}\exp\left(-\frac{qV_{s}}{2kT}\right)$$

where $C_{s}$ is the semiconductor space charge layer capacitance, *V*_s_ is the surface potential across the space charge layer of GaN, $\varepsilon_{s}$ is the permittivity of the semiconductor, $\varepsilon_{0}$ is the permittivity of the vacuum, $L_{D}$ is the Debye length, k is the Boltzmann constant, T is the absolute temperature, q is the unit electronic charge.

**Figure 6:**
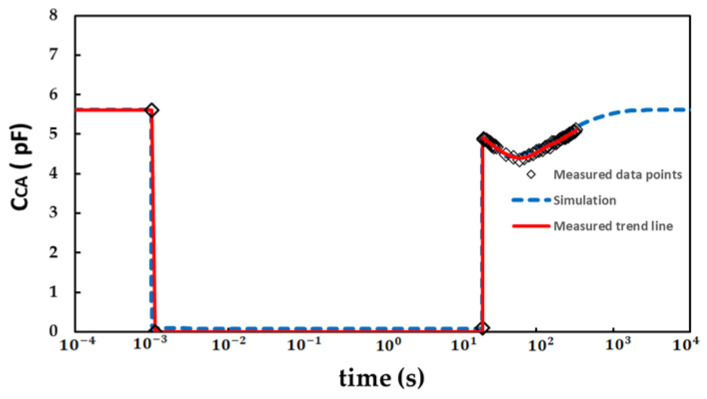
Dynamic of measurement and simulations.

The transient measurement was carried out on GaN SBDs with L_AC_ = 6 μm. At the first phase (10^−4^ s to 10^−3^ s), the initial C_CA_ (measured with V_AC_ = −1.8 V, f = 1 MHz) was measured. At the stress phase (10^−3^ s to 20 s), the stress voltage V_AC_ was set to be −150 V. At the recovery phase, the C_CA_ was taken immediately after stressing with a delay time of 100 ms. Measured and simulated results with the model mentioned in the Section 2 are summarized in Figure 6 with solid and dotted lines, respectively. In the simulation, in order to improve the convergence of simulation model, we set the anode to zero potential and applied positive bias to the cathode to achieve the effect of reverse bias. In the simulation, the C_CA_ gradually decreased at the first 20 s and gradually increased after 20 s and returned to the initial state. The measured result is in concordance with the simulation model.

**Figure 7:**
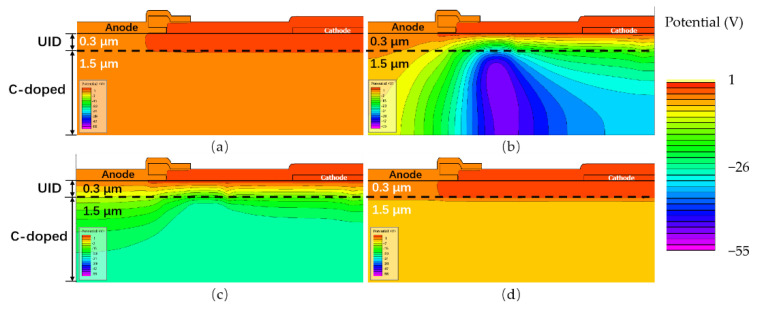
The potential distribution at different timings. (**a**) t_0_: the initial state without electrical stress; (**b**) t_1_: 1 s after the electrical stress; (**c**) t_2_: 20 s after the electrical stress; and (**d**) t_3_: 10,000 s after the electrical stress.

Figure 7 shows the potential distribution diagram at different timings. In Figure 7b, the potential in the buffer layer reduces significantly after the reverse stress. In Figure 7c,d, the potential extremum is gradually lowered and recovered after a sufficient time delay.

**Figure 8:**
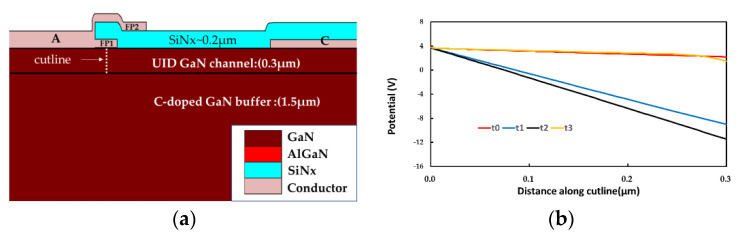
Schematic of (**a**) the cutline location; (**b**) the potential at different timings.

Figure 8a presents the location of the cutline, which is in the UID region below the center of FP1. Figure 8b shows the changing trend of potential, and the potential difference reaching the maximum value at 20 s (t_2_). According to Equation (1), we could safely conclude that C_CA_ reaches the minimum value at 20 s (t_2_).

**Figure 9:**
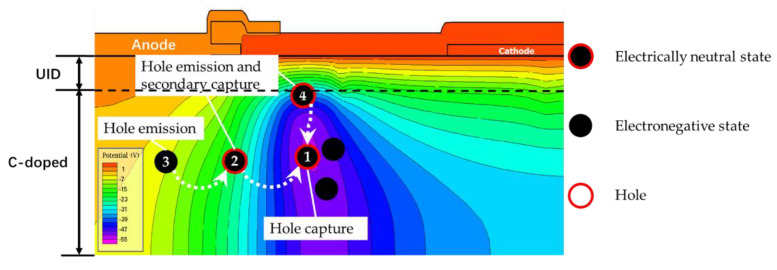
Schematic diagram of secondary capture process.

Figure 9 shows the potential distribution measured at 1 s after the electrical stress was removed. The presence of electronegative states at position 1 results in its lower potential. The denser potential lines showing the higher electric field in the vicinity would promote the acceptor traps in the vicinity to still have a higher ionization rate, even after the removal of stress. The acceptor traps at positions 2 and 4 ionize and release the holes captured by the electronegative state at position 1 under the action of potential difference. The electronegative state at position 1 captures the hole and restores the electrically neutral state, thus increasing the negative potential value. The ionized acceptor traps at position 2 will release holes and turn into an electronegative state, but the ionized acceptor traps at position 2 will capture the hole released by the acceptor traps at position 3 and restore the electrically neutral state. The continuously ionized and captured acceptor defect at position 2 could be viewed as “secondary capture”. The acceptor traps near position 3 are continuously ionized, while the holes released are captured by the electronegative state at position 2. However, no hole can restore the electric neutrality at position 3. Therefore, a large number of acceptor traps at position 3 would become electronegative, thus lowering the electric potential at position 3. Through this process, the potential at position 1 will continue to levitate, while the potential at position 3 will continue to fall.

As time goes by, the potential difference between position 3 and position 1 reduces to a level with no allowance for the “secondary capture” of the acceptor traps at position 2. The potential at position 1 no longer decreases. It occurs at the time node of about 20 s after the removal of electrical stress in simulation. Afterwards, the overall potential in the buffer layer increases and thus the potential difference in the UID-GaN gradually decreases and eventually returns to its initial state. According to our previous analysis, the change of potential difference in UID-GaN would result in capacitance variation. The minimum value of capacitance appears at about 20 s after the removal of the electrical stress, which is consistent with the trend of potential change.

**Figure 10:**
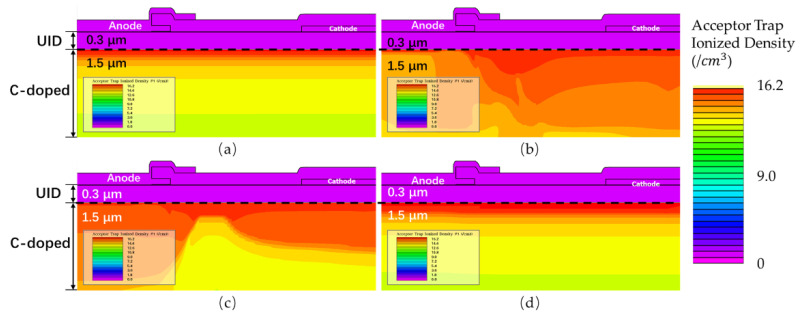
The acceptor trap ionized at different times. (**a**) t_0_: the initial state; (**b**) t_1_; (**c**) t_2_; and (**d**) t_3_.

Figure 10 shows the acceptor trap ionized distribution diagram at different times. In Figure 10b, the ionization concentration of acceptor traps reaches its maximum near the position of lowest potential. In Figure 10c, the ionization concentration is higher at position 3 in Figure 9. In Figure 10d the acceptor trap ionization is gradually lowered and recovered after a sufficient time delay. These observations are consistent with our analysis of the process of “secondary capture”.

## 4. Conclusions

Capacitance collapse characterization on a lateral AlGaN/GaN SBD was successfully carried out with a modified AccoTEST STS8200 tester. C-V characteristics and dynamic capacitance well matched the simulations from Silvaco TCAD. Based on our well-verified model, the variation of electric potential and the ionization of acceptor traps in the device were studied and analyzed in detail. The relationship between the electric potential and acceptor traps was simulated and discussed by varying the capture and emission of the acceptor traps. The “secondary capture” effect resulted in different variation levels of the acceptor ionization, leading to various potential changes and subsequently the capacitance change of the device. This study of SBD devices can be extended to the study of Miller capacitance of HEMT devices.

## Figures and Tables

**Table 1:** Parameters utilized in the simulation.

Parameters	Value	Unit
Schottky metal work function	4.65	eV
Polarization charge density in the access region	$1.35\times 10^{13}$	cm^−2^
Polarization charge density in the electrode region	$2.5\times 10^{12}$	cm^−2^
C-doping concentration in the buffer	$2\times 10^{17}$	cm^−3^
E_TA_ of the acceptor trap in the C-doped buffer	Ev + 0.95	eV
E_TD_ of the donor trap in the UID-GaN channel	Ec − 1.05	eV
Electron capture cross sections	$1\times 10^{-13}$	cm^2^
Hole capture cross sections	$1\times 10^{-13}$	cm^2^
AC frequency	$1\times 10^{6}$	Hz
Ramp time from stress to capacitance test	10	μs

## Data Availability

Not applicable.
